# Nanopore sequencing approach for immunoglobulin gene analysis in chronic lymphocytic leukemia

**DOI:** 10.1038/s41598-021-97198-3

**Published:** 2021-09-03

**Authors:** Crescenzio Francesco Minervini, Cosimo Cumbo, Immacolata Redavid, Maria Rosa Conserva, Paola Orsini, Antonella Zagaria, Luisa Anelli, Nicoletta Coccaro, Giuseppina Tota, Luciana Impera, Elisa Parciante, Francesco Tarantini, Annamaria Giordano, Giorgina Specchia, Pellegrino Musto, Francesco Albano

**Affiliations:** 1 Department of Emergency and Organ Transplantation (D.E.T.O.), Hematology and Stem Cell Transplantation Unit, University of Bari “Aldo Moro”, 70124 Bari, Italy; 2 School of Medicine, University of Bari “Aldo Moro”, 70124 Bari, Italy

**Keywords:** Chronic lymphocytic leukaemia, Immunogenetics, Bioinformatics, Next-generation sequencing

## Abstract

The evaluation of the somatic hypermutation of the clonotypic immunoglobulin heavy variable gene has become essential in the therapeutic management in chronic lymphocytic leukemia patients. European Research Initiative on Chronic Lymphocytic Leukemia promotes good practices and standardized approaches to this assay but often they are labor-intensive, technically complex, with limited in scalability. The use of next-generation sequencing in this analysis has been widely tested, showing comparable accuracy and distinct advantages. However, the adoption of the next generation sequencing requires a high sample number (run batching) to be economically convenient, which could lead to a longer turnaround time. Here we present data from nanopore sequencing for the somatic hypermutation evaluation compared to the standard method. Our results show that nanopore sequencing is suitable for immunoglobulin heavy variable gene mutational analysis in terms of sensitivity, accuracy, simplicity of analysis and is less time-consuming. Moreover, our work showed that the development of an appropriate data analysis pipeline could lower the nanopore sequencing error rate attitude.

## Introduction

The evaluation of the somatic hypermutation (SHM) of the clonotypic immunoglobulin heavy variable (IGHV) gene has become essential in the therapeutic management of chronic lymphocytic leukemia (CLL) patients. In fact, it has been shown to be a robust prognostic marker, stable over time and independent of other clinical and biological parameters, including the disease progression^1^.

The gold standard method for determining the SHM status is performed in two steps: (a) clonality detection by PCR and capillary electrophoresis (CE); (b) Sanger sequencing (SS) of the clonotypic IGHV gene. The sequencing result is then evaluated for its deviation compared with the closest matched germline IGH gene, using the 2% threshold to discriminate unmutated from mutated status^1,2^.

Despite the European Research Initiative's efforts in CLL (ERIC) to promote good practices and standardized approaches, this assay is still not uniformly performed in many clinical laboratories due to its limitations, that include labor-intensiveness, technical complexity, and limited scalability.

The recent availability of next-generation sequencing (NGS) technologies offers the chance to develop more standardized methods to unambiguously determine the individual clonal sequences and their relative proportions^3–5^. The use of NGS for SHM analysis has been widely tested, showing comparable accuracy, but distinct advantages of NGS included: the possibility to operate in batch mode, low costs (only running in batch mode), direct clone determination, and a greater sensitivity allowing more than one dominant clonal IGH rearrangement, that has been reported in almost 25% of CLL patients, to be identified^6^. On the other hand, the adoption of NGS requires a high sample number (batch running) to be economically convenient, leading to a longer turnaround time. Moreover, data analysis and interpretation are more complicated and not yet standardized^6^.

MinION is the smallest and cheapest NGS third-generation platform currently available, based on nanopore sequencing (NS)^7^. It can produce two different throughput ranges (1–2 Gb up to 40 Gb) depending on the flowcell used, and library preparation is easy and rapid compared with the currently adopted technologies^8–11^. These features make NS technology an excellent candidate for adoption in small labs, even if this approach still suffers from a high error rate, incompatible with clinical demands. On the other hand, this weakness has been considerably reduced thanks to the improvements in sequencing chemistry, the introduction of new base-calling algorithms, and the use of post-sequencing correction tools^8–10,12,13^. Therefore, in the CLL SHM analysis context, NS could offer a convenient alternative in terms of costs, throughput scalability, and ease of use.

Our report presents data on NS analysis for the SHM evaluation in CLL patients, as compared with the SS method. We developed a bioinformatic pipeline for sequence assembly, correction, clonality assessment, and mutational status analysis.

## Results

### SHM analysis by SS

All samples were evaluated by SS with the leader primers, as indicated by the ERIC recommendations. A second-round evaluation was performed with FR1 primers to ensure a more accurate comparison with the data generated by NS analysis. Final SHM analysis of the 36 samples produced the following results: 27 single (12 unmutated, 15 mutated), and 9 double VDJ recombination (7 productive/unproductive with concordant status, 2 double productive with concordant status). Sample #29 was borderline (V-REGION identity % = 97.80). For all cases it was possible to determine the mutational status, and no difference was detected between the two types of primers (Supplementary Table 2).

### SHM analysis by MinION sequencing

**Figure 1:**
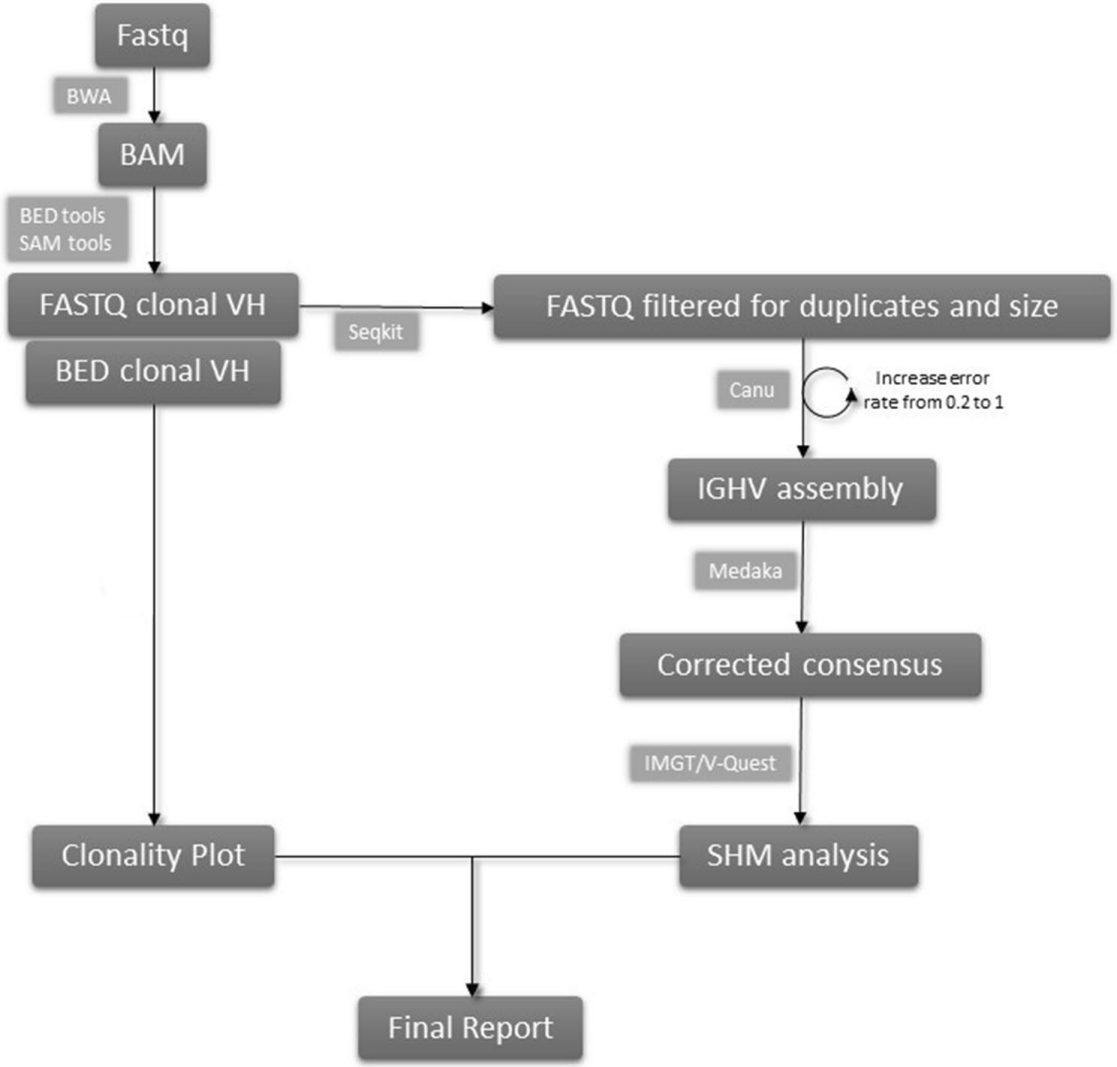
Schematic representation of the NanoIg pipeline.

A total of three runs was performed, employing three libraries of 12 patients, and three different flongle flowcells, with 74, 65, and 61 active pores, respectively. Each run lasts 24 h and produced respectively: 855.897, 860.106, and 1.251.284 reads. After base-calling, each run generated more than 0.4Gbases. Plotting the distribution of read lengths, we observed a prevalent peak around 0.5 Kb, in agreement with our amplicon size range. Basecalled and demultiplexed data were then analyzed on our NanoIg Pipeline (Fig. 1 and “Material and methods”). Each analysis lasts around 12 h on Intel® CORE™ i7-7700 K CPU @420 GHz 16.0 GB RAM depending on the total of reads.

### Comparative evaluation

**Figure 2:**
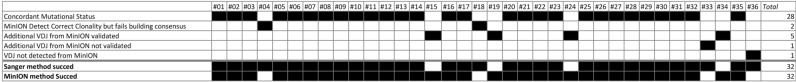
Summary of results considering as primary goal the determination of the correct mutational status. The success of the analysis is indicated with the black box. Besides the concordant status category, other categories represent differences both in clonality assessment and consensus building.

The SHM analysis results from both methods were compared. Comparative results are summarized in Fig. 2 (details are reported in Supplementary Table 3).

Comparing the final mutational status between SS and NS analysis for each sample (first row Fig. 2), among the 36 samples, 28 (78%) were concordant.

Eight samples (22%) showed a different final mutational status between the two methods. NS analysis on cases #4 and #18 did not produce a VDJ consensus even though clonal VH genes were correctly reported in the clonality plot (respectively V4-39/D2-2/J6 and V3-30/D5-18/J6). In addition, for NS analysis on case #36 the productive rearrangement was not detected (V1-69/D3-16/J3) in the clonality plot or generate a productive consensus.

On the other hand, NS analysis for samples #15, #19, #24, #33, and #34 showed additional VDJ recombinations that were not detected by SS methodology.

**Figure 3:**
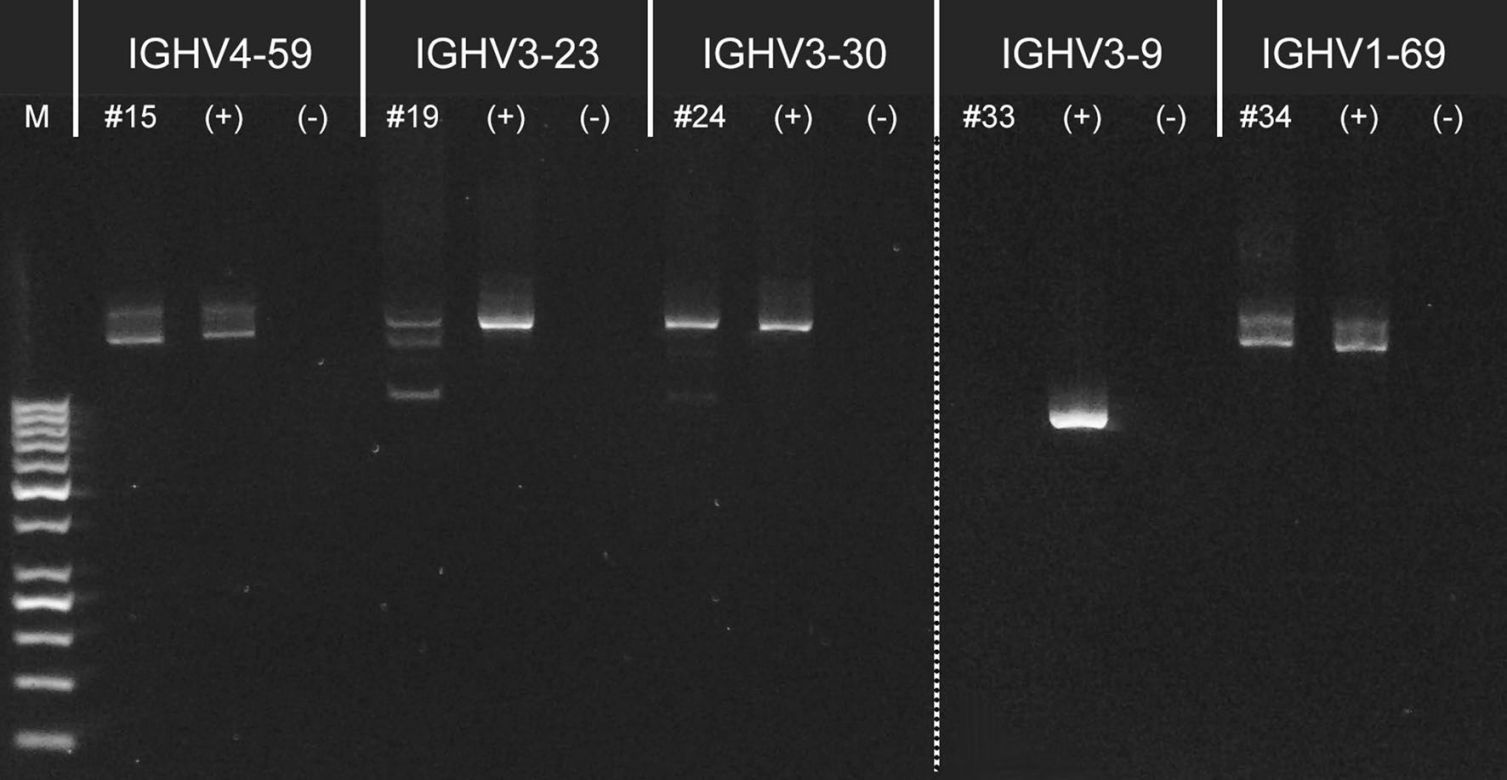
VH specific PCR validation for samples #15, #19, #24, #33 and #34. For each specific VH to be validated, the same PCR is performed on the sample to validate positive (+) and negative (−) controls. Positive controls were samples previously determined for the specific VDJ recombination; instead, negative controls were samples previously tested with different VDJ recombinations. The dotted line delimits two different gels (original images are provided as Supplementary Fig. 4 and Supplementary Fig. 5).

To establish the correct mutational state of this samples the additional VDJs rearrangements found, were validated by specific VH-PCR (Fig. 3 and “Discordant data validation” in Material and methods). For samples #15, #19, #24, and #34 the specific VH-PCR showed the actual existence of the additional recombinations not previously detected by SS method, whereas in case #33 the validation attempt was unsuccessful.

Moreover, two patients (#3 and #13) with concordant status showed differences in the number of clonal recombinations detected. In detail, sample #3 showed IGHV4-34 recombination, detected only by NS, whereas case #13 showed an additional IGHV4-34 recombinant that NS did not detect. In both cases, this did not affect the mutational status evaluation and therefore are not reported as discordant in Fig. 2.

In summary, considering the results of validations and the relative changes in the final mutational status, the percentage of successful analysis for both the SS and NS methods was 88.8% (Fig. 2 last two rows).

**Figure 4:**
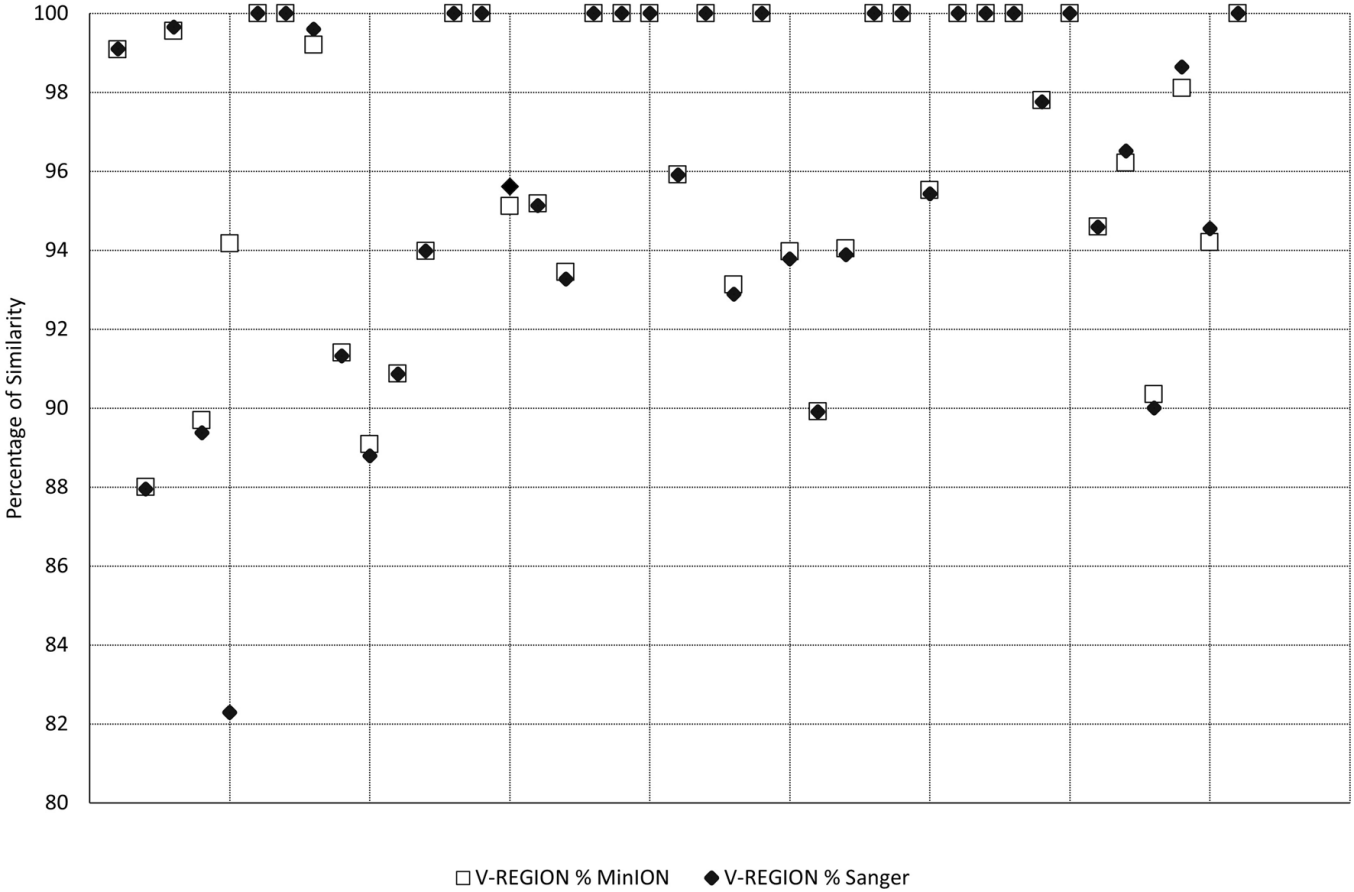
Comparison between the VH region percentage of similarity as obtained from IMGT/V-Quest analysis. Data from SS and MinION for each specific VH and sample are compared. VH that were found only in SS analysis or MinION analysis are excluded from the graph.

A side-by-side comparison of the percentage of VH genes similarity found in both methods was further performed, as reported in Fig. 4. Excluding IGHV3-23 of case #5, the difference for the other cases was always within 0.5%.

## Discussion

NGS has brought in a new era in medical diagnostics, and IGHV analysis is following this trend. Nowadays, there is a growing demand for shifting the standard IGHV gene SHM analysis in favor of NGS. However, this change must take into account the financial status of laboratories with limited resources and the absence of technical aspects and data interpretation standardization. Regarding the latter point, the ERIC IG network and EuroClonality-NGS Working Group are currently working on this. Therefore, NGS will probably become the IGHV gene SHM assessment method of choice, but traditional SS remains the standard method today. The NS technology has already proven to be a valuable tool in analyzing often mutated genes in CLL^9,10^. In the IGHV analysis context, our report results demonstrate the non-inferiority of the NS approach to the standard, as in our series, the NS analysis provides equal or better information than SS. In 5 (13.8%) cases, NS revealed multiple productive IGHV subclones. The FR1 and leader primers consensus nature does not allow the best annealing, and this event can further be compromised when the SHM falls in the annealing region. To improve the PCR efficiency, we designed primer pairs annealing in intergenic regions, upstream of the J6 gene and downstream of the specific VH, that would not be involved in the somatic hypermutations events. In this way, we validated all five additional IGH rearrangements (Fig. 3).

Notably, some shows inconsistency between clonality and the consensus IMGT/V-Quest analysis result (Supplementary Figs. 1–3). The reasons for this inconsistency stem essentially from the different references used for IMGT/V-QUEST and our clonality analysis. The IMGT/V-QUEST reference directory is constituted by sets of sequences that contain the V-REGION, D-REGION, and J-REGION alleles, isolated from the functional and ORF allele IMGT reference sequences. Sequences are obtained from clones, subclones or PCR, and do not apply to sequences obtained directly from genomic DNA (http://www.imgt.org/vquest/refseqh.html#VQUEST). In our analysis, instead, we mapped reads on GRCh38/hg38 human genome reference. Moreover, the error-prone nature of reads from nanopore sequencing generates reads dispersion during alignment due to the similarity within the same VH family. Furthermore, consensus generation from NanoIG pipeline includes error correction and allow the correct VDJ rearrangement detection from IMGT/V-Quest analysis.

The circumstance of multiple productive subclones has already been reported in IGHV analysis by NGS^6^, and it could contribute to better define the CLL biological and prognostic heterogeneity.

NS analysis reported for #04, #18, #13 and #36 was missing an IGHV recombination compared to SS analysis. This loss for cases #04 and #18 occurred during consensus building because the clonality plot reported the same clonotypic variable genes found in the standard method (Supplementary Figs. 1 and 2). The principal reason for consensus-building failure was the various data filtering steps in the pipeline. Whereas in cases #13 and #36, lacking recombination was due to a failure in the amplification step (Supplementary Figs. 2 and 3).

. The use of FR1 primers to build the library for NS analysis represents a major critical point of our work. ERIC guidelines recommend using leader primers for the VDJ amplification and subsequent sequencing, but this primer set is known to have low amplification efficiencies^14,15^. Moreover, it is well-documented that the advantage of using leader primers is limited to the fact that this strategy could change the mutational status of a small fraction (estimated as 0.4%) of the CLL patients^15^. In fact, in our series of 36 CLL patients, the IGHV mutational status resulted the same when using leader and FR1 primer sets. In a previous report, FR1 primers were employed for NGS analysis because the leader would have generated a mean length of amplified products close to exceeding the expected read length range^16^. In the context of NS, the long-read sequencing analysis could have benefited from using the leader primers to increase amplicon length. Unfortunately, the leader primers were unsuccessful when used in multiplex to prepare the library for the NS analysis. Even if it is unlikely that the use of FR1 primers in the NS approach could have jeopardized the results in our set, a future clinical application of this method needs to be improved to include the Leader region in the sequencing.

Comparative analysis of the VH genes percentage of similarity revealed that the sequencing results were almost overlapping between the SS and NS, except in one case (case #5). Nevertheless, in the latter case, the IGHV mutational status was in agreement between the two methods. Therefore, VH genes comparative analysis further confirms the accuracy of NS data generation.

In conclusion, here we show that in IGHV molecular analysis the NS approach is reliable, is not inferior to SS and, enables the detection of small subclones. Our NanoIG pipeline proved to be a helpful tool for improving the sequencing data quality generated by NS. Due to its practicality and low cost (twelve samples were analyzed in 4–5 days and the estimated cost per sample was around 20 Euros), NS technology is potentially an ideal candidate to lead the evaluation of the IGHV mutational status towards a new standard of analysis.

## Materials and methods

### Patients

Thirty-six newly diagnosed CLL patients were included in this study. Genomic DNA was extracted from peripheral blood using the QIAamp DNA Blood Mini Kit (Qiagen); the DNA concentration and purity were checked using the Qubit 2.0 fluorometer (Life Technologies) and Nanodrop UV–Vis spectrometer (Thermo Fisher Scientific). The local ethics committee of “Azienda Ospedaliero Universitaria Policlinico Bari” (University of Bari) approved the study. Informed consent was obtained from all patients before study inclusion, in accordance with the Declaration of Helsinki. Patients' records/information were anonymized and de-identified before analysis.

### IGHV analysis

According to ERIC recommendations^2^, the IGHV region was amplified using leader primers for clonality and SHM assessment. To better compare data and improve the polymerase-chain-reaction (PCR) performance in the subsequent NS library preparation, we decided to reevaluate all samples with FR1 consensus primers from the European BIOMED-2 collaborative study^17^.

### SS analysis

For each case, two multiplex PCRs were performed using Platinum Taq DNA Polymerase (Invitrogen), with 50 ng of gDNA, labeled V-gene primers, and the J-gene primer^17^, in a final volume of 25 uL. The amplification results (0.5 μl) were prepared for fragment analysis by CE according to the Seqstudio genetic analyzer user guide. For the SHM assessment, the clonal rearrangement was then reamplified in a new PCR (under the same conditions), loaded on a 2% agarose gel, purified with the QIAquick PCR Purification Kit (Qiagen), and prepared for SS. The IMGT/V-QUEST^18,19^ and ARResT/AssignSubsets^20^ tools were used for the IGHV identity assessment and stereotypic subset identification.

### NS analysis

#### Library preparation and sequencing

For each case, a multiplex PCR^17^ was performed. Because of a high amplification failure rate of leader primers in multiplex strategy (about 30% in our hands), we used the FR1 primers instead.

PCR products were purified with the QIAquick PCR Purification Kit (Qiagen), and the DNA concentration and purity were measured with a Qubit 2.0 fluorometer (Life Technologies) and Nanodrop UV–Vis spectrometer (Thermo Fisher Scientific). In accordance with the ONT Native barcoding genomic DNA protocol (EXP-NBD104), amplicons (350 ng) were end-prepared and barcoded with the ligation of ONT Native Barcodes (NB01–NB12). Equimolar amounts of each barcoded amplicon were then pulled (12 samples each pool). Following the Ligation Sequencing Kit (SQK-LSK109) protocol, 25 ng of the pooled amplicons were prepared for sequencing. After the Platform QC run and the priming of the Flongle flowcell, the sequencing mix was loaded, and a 24-h sequencing run protocol was started (MinIONflowcell: FLO-FLG001).

#### NanoIG pipeline

Fast5 files produced from sequencing were basecalled, filtered by quality, demultiplexed by MinKnow software, and the resulting fastq pass reads were analyzed on our NanoIG pipeline (see “Results”). The pipeline is available at https://github.com/eziominervini/NanoIg.

The NanoIG pipeline performs both clonality and SHM analysis (Fig. 1) on nanopore data. Reads are first mapped on chromosome 14, and the depth of sequence on immunoglobulin variable region genes is calculated. The clonotypic variable gene is determined from coverage values after filtering. In this study, we set the coverage threshold at 500X. Genomic coordinates of clonal VH genes were then used to select the reads for subsequent VDJ consensus building.

The VDJ consensus is built using canu assembler^21^ and medaka for correction (https://nanoporetech.github.io/medaka/index.html). Due to the MinION error rate, the first assembly performed by canu is set at a relatively low initial value for acceptable error rate, that is increased until the maximum value or until the assembly achievement. Before medaka correction, assemblies are filtered by supporting reads number.

Final consensus sequences are then used to interrogate IMGT/V-Quest, and results are downloaded. The report is produced by plotting clonality results and tabulating IMGT/V-Quest results of all samples.

### Discordant data validation

Alternative PCRs were designed using the same J-gene primer as before^17^ and specific V-gene primers mapping in the intragenic region downstream of the V-gene to clarify discordant cases (Supplementary Table 1). The same PCR conditions were applied as previously described.

## Supplementary Information

Supplementary Information.

## Data Availability

This study sequencing data have been submitted to the National Center for Biotechnology Information (NCBI) Short Read Archive (https://www.ncbi.nlm.nih.gov/sra/)under PRJNA731004.
