# Supplementary material for: Nanopore sequencing approach for immunoglobulin gene analysis in chronic lymphocytic leukemia
Source: Sci Rep. 2021 Sep 3;11:17668. doi: 10.1038/s41598-021-97198-3 (PMC8417258; doi:10.1038/s41598-021-97198-3)
Supplement: Supplementary file 1 — Supplementary Information. [file 41598_2021_97198_MOESM1_ESM.pdf]

Supplementary fig1 Clonality analysis results from MinION run1.

Supplementary fig2 Clonality analysis results from MinION run2.

Supplementary fig3 Clonality analysis results from MinION run3.

Supplementary fig4 Gel electrophoresis of VH specific PCR for samples #15, #19, #24

Supplementary fig5 Gel electrophoresis of VH specific PCR for samples #33 and #34

Supplementary table 1 VH specific PCR primers

Supplementary table 2 Summary and comparison of IGVH mutational analysis performed with Leader Primers sequencing and FR1 Primers by Sanger Sequencing.

Supplementary table 3 Summary and comparison of IGVH mutational analysis from IMGT/V-Quest performed with Sanger sequencing and MinION data.

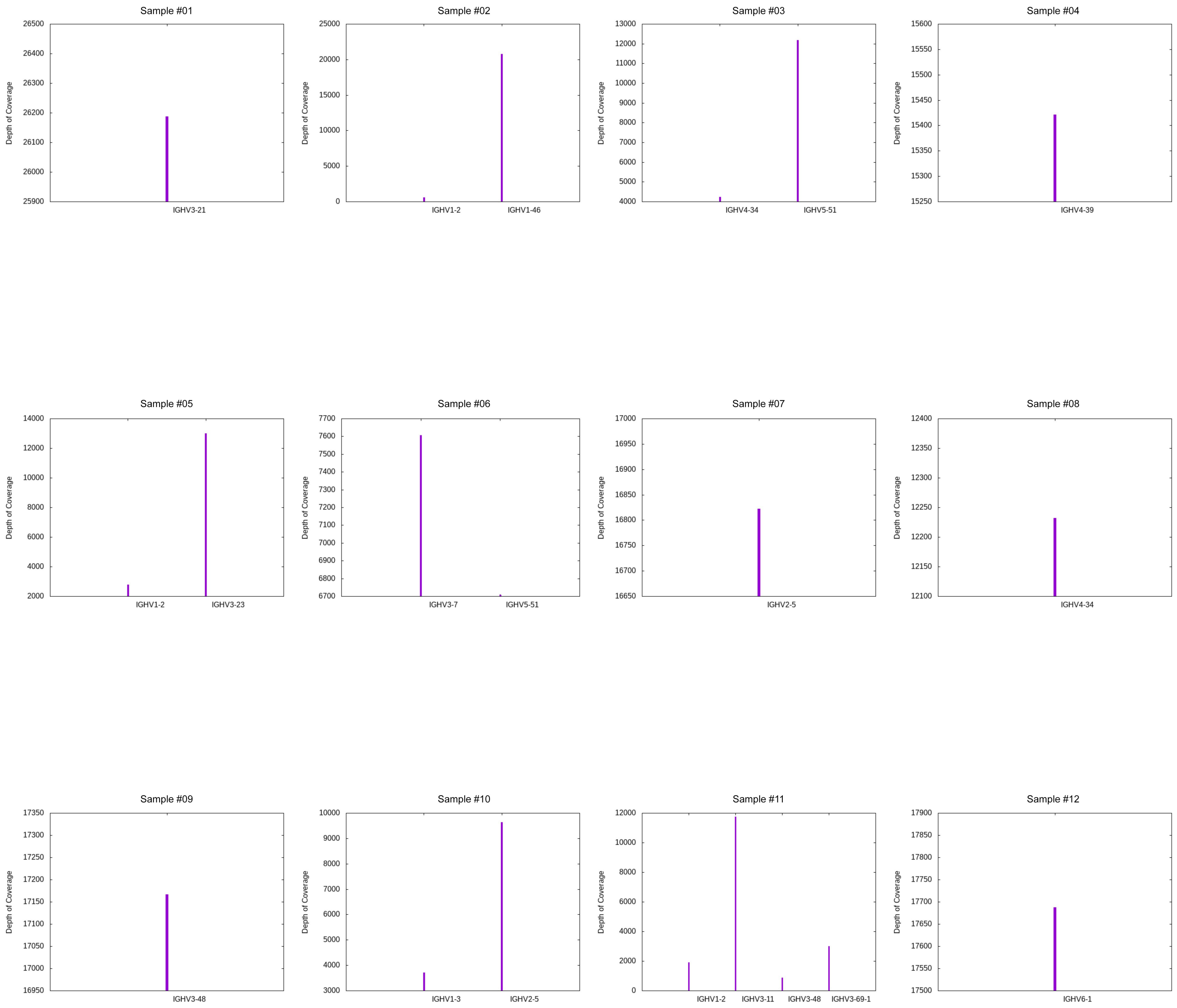

Supplementary fig1

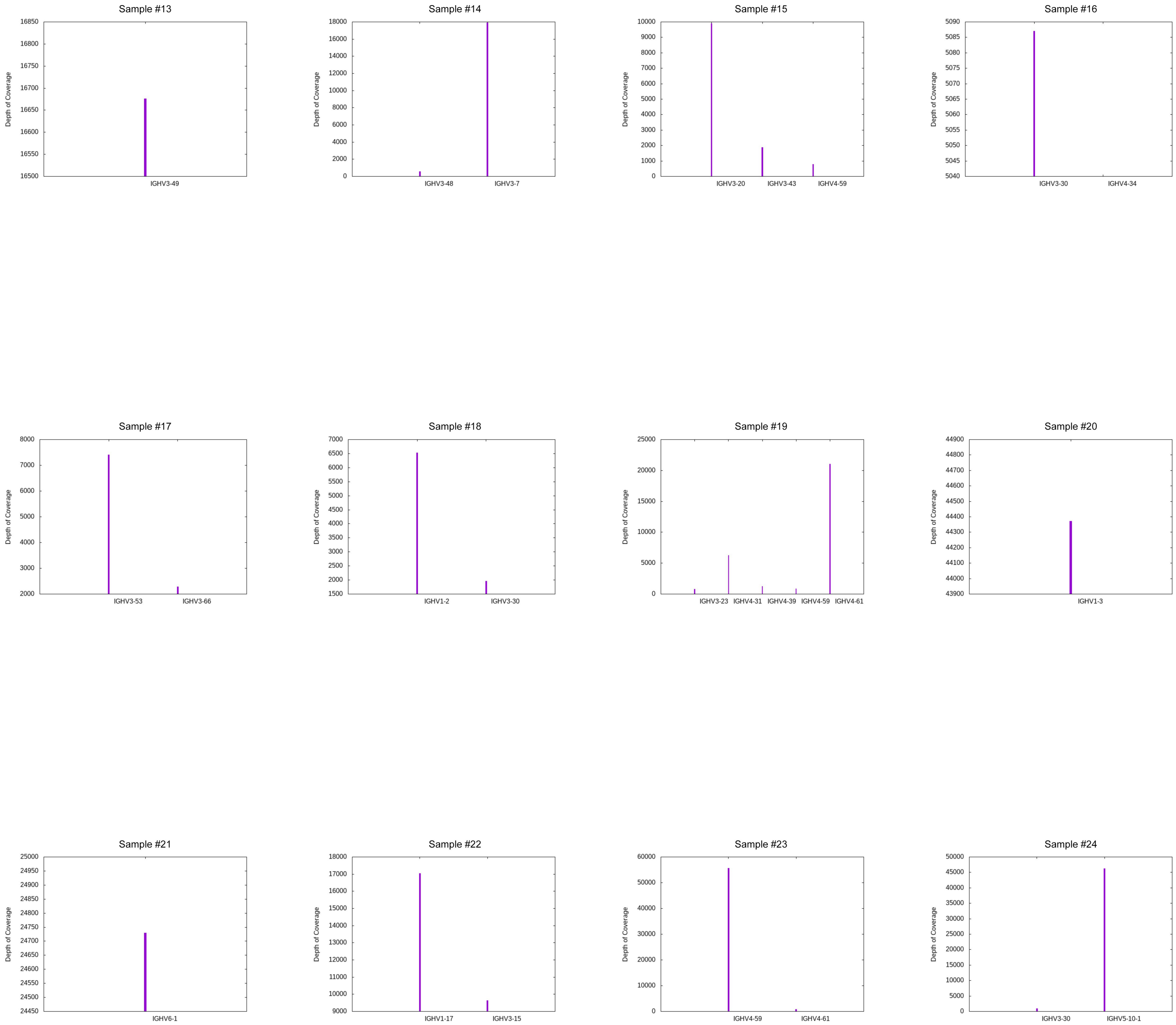

Supplementary fig2

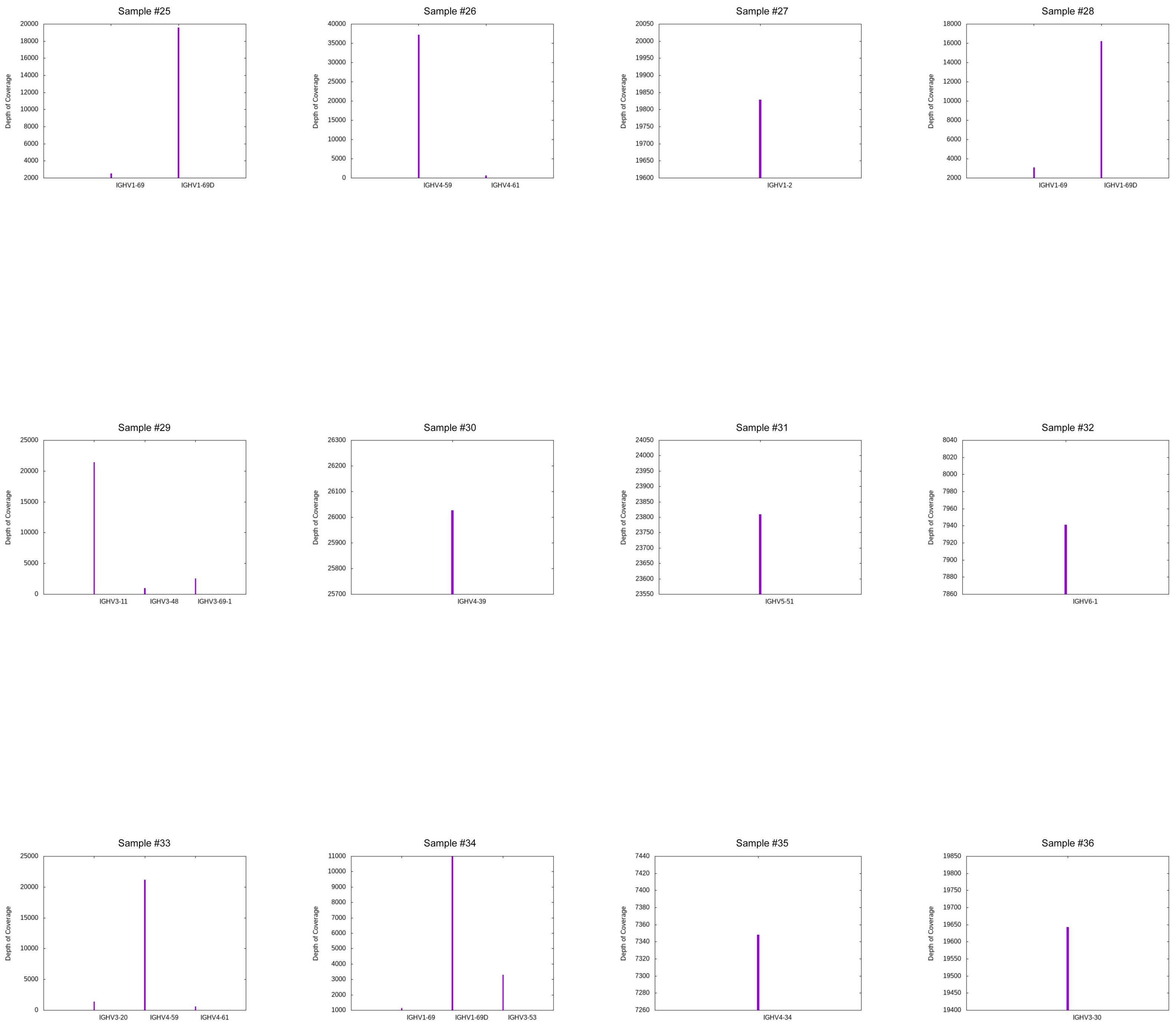

Supplementary fig3

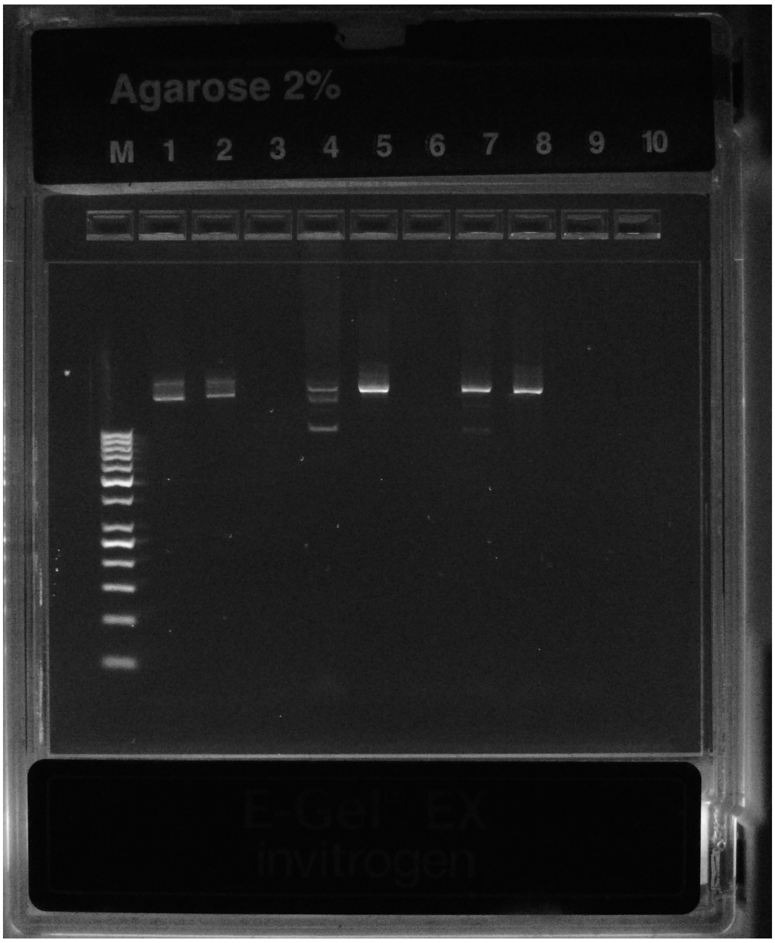

Supplementary fig4

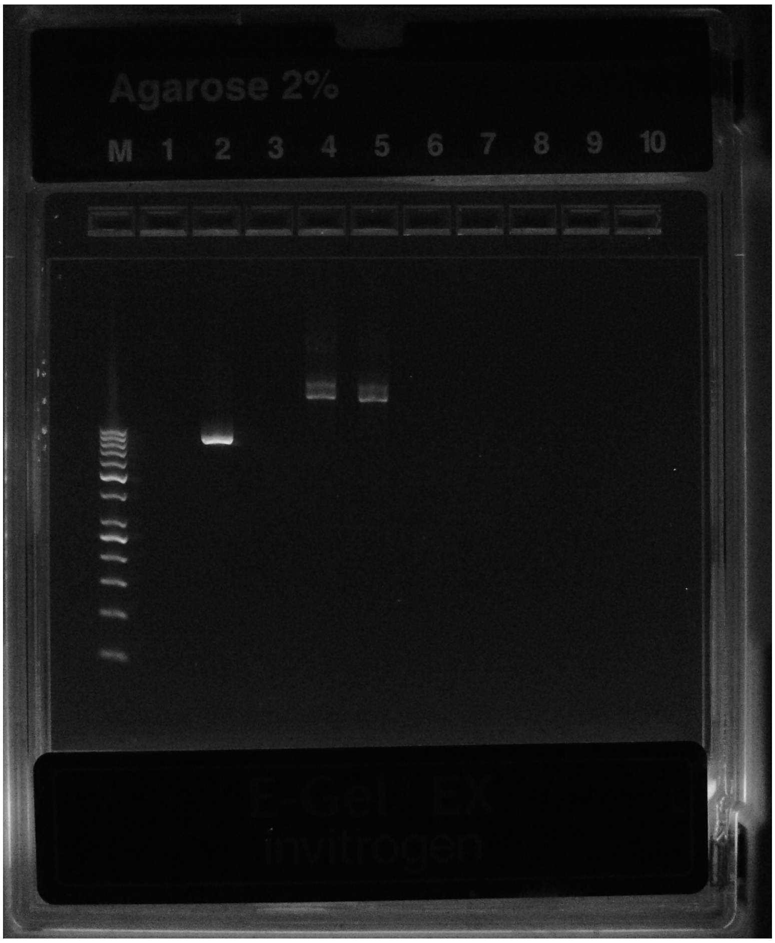

Supplementary fig5

Supplementary table 1

Primer Name	Sequence
UPJ	acctgcaatgctcaggaaac
IGHV3-23_R	TGCTCTGAGCCCCACTATCT
IGHV3-30_R	CAGCCTCTCCTCAGATGTCC
IGHV1-69_R	TCAGGCTTTGAGCTGTGAAA
IGHV3-9_R	AGAGGCATCCCTTCCAGAAC
IGHV4-59_R	GGAAACCACCACACATTTCC

Supplementary table 2

Patient	V-GENE and allele	V-DOMAIN Functionality	V-REGION identity % (nt)	J-GENE and allele	D-GENE and allele	CDR-IMGT lengths	AA JUNCTION	Mutational Status
#01	IGHV3-21	productive	98.61% (284/288 nt)	IGHJ4	IGHD3-22	[8.8.12]	CARPSDDSSMFDYW	Unmutated
	IGHV3-21	productive	99.10% (221/223 nt)	IGHJ4	IGHD3-22	[8.8.12]	CARPSDDSSMFDYW	Unmutated
#02	IGHV1-46	productive	89.58% (258/288 nt)	IGHJ4	IGHD1-26	[8.8.12]	CARRSNTKGDFDYW	Mutated
	IGHV1-46	productive	87.95% (197/224 nt)	IGHJ4	IGHD1-26	[8.8.12]	CARRSNTKGDFDYW	Mutated
#03	IGHV5-51	productive	99.65% (287/288 nt)	IGHJ6	IGHD6-19	[8.8.19]	CARRSDPNGGWSYYYYGMDVW	Unmutated
	IGHV5-51	productive	99.55% (221/222 nt)	IGHJ6	IGHD6-19	[8.8.19]	CARRSDPNGGWSYYYYGMDVW	Unmutated
#04	IGHV4-39	productive	100.00% (247/247 nt)	IGHJ6	IGHD2-2	[10.7.22]	CARDRGGYCSSTSCYYYYYGMDVW	Unmutated
	IGHV4-39	productive	100.00% (226/226 nt)	IGHJ6	IGHD2-2	[10.7.22]	CARDRGGYCSSTSCYYYYYGMDVW	Unmutated
#05	IGHV1-2	unproductive	91.32% (263/288 nt)	IGHJ4	IGHD1-26	[8.8.9]	SFSNSENFLLR	Mutated
	IGHV3-23	productive	83.40% (259/288 nt) [80.91% (252/288 nt)]	IGHJ4	IGHD5-24	[8.8.10]	CAKGRDGYIHRW	
	IGHV1-2	unproductive	89.38% (202/226 nt)	IGHJ1	IGHD1-26	[8.8.9]	SFSNSENFLLR	Mutated
	IGHV3-23	productive	82.30% (186/226 nt) [80.08% (181/226 nt)]	IGHJ4...	IGHD5-24	[8.8.10]	CAKGRDGYIHRW	
#06	IGHV3-7	unproductive	100.00% (288/288 nt)	IGHJ4	IGHD6-19	[8.8.X]	CARLLVGPSDKGIAVAD#FDYW	Unmutated
	IGHV5-51	productive	100.00% (288/288 nt)	IGHJ4	IGHD3-22	[8.8.14]	CARRQTWTLVPHYDYW	
	IGHV3-7	unproductive	100.00% (215/215 nt)	IGHJ4	IGHD6-19	[8.8.X]	CARLLVGPSDKGIAVAD#FDYW	Unmutated
	IGHV5-51	productive	100.00%(228/228 nt)	IGHJ4	IGHD3-22	[8.8.14]	CARRQTWTLVPHYDYW	
#07	IGHV2-5	productive	99.31% (289/291 nt)	IGHJ5	IGHD3-3	[10.7.23]	CAHTLQTYYDFWSGQPRRWGWFDPW	Unmutated
	IGHV2-5	productive	99.60% (248/249 nt)	IGHJ5	IGHD3-3	[10.7.23]	CAHTLQTYYDFWSGQPRRWGWFDPW	Unmutated
#08	IGHV4-34	productive	93.31% (265/284 nt)	IGHJ4	IGHD2-21	[8.7.16]	CARGNCGGDCYFSPFDSW	Mutated
	IGHV4-34	productive	91.32% (200/219 nt)	IGHJ4	IGHD2-21	[8.7.16]	CARGNCGGDCYFSPFDSW	Mutated
#09	IGHV3-48	productive	91,32% (263/288 nt) [90,97% (262/288 nt)]	IGHJ4	IGHD1-26	[8.8.7]	CARGLGDYW	Mutated
	IGHV3-48	productive	88.79% (198/223 nt) [88.34% (197/223 nt)]	IGHJ4	IGHD1-26	[8.8.7]	CARGLGDYW	Mutated
#10	IGHV1-3	productive	91.99% (264/287 nt)	IGHJ4	IGHD6-6	[8.8.17]	CARDWGVSSSPKWEHFDYW	Mutated
	IGHV2-5	productive	94.16% (274/291 nt)	IGHJ1	IGHD6-6	[10.7.14]	CAHRDYSTSSGYFQHW	
	IGHV1-3	productive	90.87% (199/219 nt)	IGHJ4	IGHD6-6	[8.8.17]	CARDWGVSSSPKWEHFDYW	Mutated
	IGHV2-5	productive	93.98% (234/249 nt)	IGHJ1	IGHD6-6	[10.7.14]	CAHRDYSTSSGYFQHW	
#11	IGHV1-2	productive	100.00% (288/288 nt)	IGHJ6	IGHD3-9	[8.8.22]	CAREYGVDILTGYLLLCTYGMDVW	Unmutated
	IGHV3-11	unproductive	100.00% (288/288 nt)	IGHJ4	IGHD3-22	[8.8.24]	CARARVEP*PEL***W*KNPTPFDYW	
	IGHV1-2	productive	100.00% (219/219 nt)	IGHJ6	IGHD3-9	[8.8.22]	CAREYGVDILTGYLLLCTYGMDVW	Unmutated
	IGHV3-11	unproductive	100.00% (223/223 nt)	IGHJ4	IGHD3-22	[8.8.24]	CARARVEP*PEL***W*KNPTPFDYW	
#12	IGHV6-1	productive	95.62% (284/297 nt)	IGHJ4	IGHD1-1	[10.9.12]	CVRDPTNWSYFDYW	Mutated
	IGHV6-1	productive	94.78% (218/230 nt)	IGHJ4	IGHD1-1	[10.9.12]	CVRDPTNWSYFDYW	Mutated
#13	IGHV3-49	productive	96,22% (280/291 nt) [95.88% (279/291 nt)]	IGHJ1	IGHD3-22	[8.9.16]	CTRSYDGSGYYPGYFLLW	Mutated
	IGHV4-34	productive	94.39% (269/285 nt)	IGHJ6	IGHD3-22	[8.7.20]	CARVSFEGSYDISGYNYYMDVW	
	IGHV3-49	productive	95.13% (215/226 nt) [94.69% (214/226 nt)]	IGHJ1	IGHD3-22	[8.9.16]	CTRSYDGSGYYPGYFLLW	Mutated
	IGHV4-34	productive	92.73% (204/220 nt)	IGHJ6	IGHD3-22	[8.7.20]	CARVSFEGSYDISGYNYYMDVW	
#14	IGHV3-7	productive	94.44% (272/288 nt)	IGHJ4	IGHD6-19	[8.8.14]	CATYRETSGWYALDSW	Mutated
	IGHV3-7	productive	93.24% (208/223 nt)	IGHJ4	IGHD6-19	[8.8.14]	CATYRETSGWYALDSW	Mutated
#15	IGHV3-9	productive	100.00% (288/288 nt)	IGHJ6	IGHD2-15	[8.8.25]	CAKEFRGGWYYFPPPSHHYYYYGMDVW	Unmutated
	IGHV3-9	productive	100.00% (223/223 nt)	IGHJ6	IGHD2-15	[8.8.25]	CAKEFRGGWYYFPPPSHHYYYYGMDVW	Unmutated
#16	IGHV3-30	productive	100.00% (288/288 nt)	IGHJ4	IGHD6-19	[8.8.12]	CARDPSSGWYLDYW	Unmutated
	IGHV4-34	unproductive	100.00% (285/285 nt)	IGHJ4	IGHD3-16	[8.7.X]	CASINDYVWGSYR*P#YYFDYW	
	IGHV3-30	productive	100.00% (223/223 nt)	IGHJ4	IGHD6-19	[8.8.12]	CARDPSSGWYLDYW	Unmutated
	IGHV4-34	unproductive	100.00% (220/220 nt)	IGHJ4	IGHD3-16	[8.7.X]	CASINDYVWGSYR*P#YYFDYW	
#17	IGHV3-53	productive	96.14% (274/285 nt) [95.79% (273/285 nt)]	IGHJ6	IGHD3-9	[8.7.21]	CAREQQDYNLSTGSYFYYGMDVC	Mutated
	IGHV3-53	productive	95.91% (211/220 nt) [95.45% (210/220 nt)]	IGHJ6	IGHD3-9	[8.7.21]	CAREQQDYNLSTGSYFYYGMDVC	Mutated
#18	IGHV1-2	unproductive	100.00% (288/288 nt)	IGHJ4	IGHD2-2	[8.8.X]	CARASCSSTSY*R#FDYW	Unmutated
	IGHV3-30	productive	100.00% (288/288 nt)	IGHJ6	IGHD5-18	[8.8.24]	CARDQDSFFHLADVDTAMSDYGMDVW	
	IGHV1-2	unproductive	100.00% (219/219 nt)	IGHJ4	IGHD2-2	[8.8.X]	CARASCSSTSY*R#FDYW	Unmutated
	IGHV3-30	productive	100.00% (223/223 nt)	IGHJ6	IGHD5-18	[8.8.24]	CARDQDSFFHLADVDTAMSDYGMDVW	
#19	IGHV4-31	productive	94.48% (274/290 nt)	IGHJ4	IGHD2-8	[10.7.11]	CAREMYGSSFDYW	Mutated
	IGHV4-31	productive	92.89% (209/225 nt)	IGHJ4	IGHD2-8	[10.7.11]	CAREMYGSSFDYW	Mutated
#20	IGHV1-3	productive	100.00% (288/288 nt)	IGHJ6	IGHD1-26	[8.8.17]	CARMYRGSYYYYYYGMDVW	Unmutated
	IGHV1-3	productive	100.00% (219/219 nt)	IGHJ6	IGHD1-26	[8.8.17]	CARMYRGSYYYYYYGMDVW	Unmutated
#21	IGHV6-1	productive	94.61% (281/297 nt)	IGHJ5	IGHD1-26	[10.9.19]	CARGGVGATERTRRKNWFDPW	Mutated
	IGHV6-1	productive	93.78% (211/225 nt)	IGHJ5	IGHD1-26	[10.9.19]	CARGGVGATERTRRKNWFDPW	Mutated

#22	IGHV1-NL1	unproductive	92,33% (265/287 nt) [91.99% (264/287 nt)]	IGHJ6	IGHD3-3	[8.8.X]	CVRDP*NAIFGVVI*#YYCGMDVW	Mutated
	IGHV3-15	productive	95.24% (280/294 nt)	IGHJ3	IGHD2-15	[8.10.11]	CARGGGGGAWGIW	
	IGHV1-NL1	unproductive	89.91% (196/218 nt) [89.45% (195/218 nt)]	IGHJ6	IGHD3-3	[8.8.X]	CVRDP*NAIFGVVI*#YYCGMDVW	Mutated
	IGHV3-15	productive	93.89% (215/229 nt)	IGHJ3	IGHD2-15	[8.10.11]	CARGGGGGAWGIW	
#23	IGHV4-59	productive	100.00% (285/285 nt)	IGHJ6	IGHD5-24	[8.7.17]	CARDGPIDTFYYYYGMDVW	Unmutated
	IGHV4-59	productive	100.00% (220/220 nt)	IGHJ6	IGHD5-24	[8.7.17]	CARDGPIDTFYYYYGMDVW	Unmutated
#24	IGHV5-10-1	productive	100.00% (288/288 nt)	IGHJ4	IGHD6-19	[8.8.13]	CARVQWLGLEYFDYW	Unmutated
	IGHV5-10-1	productive	100.00% (222/222 nt)	IGHJ4	IGHD6-19	[8.8.13]	CARVQWLGLEYFDYW	Unmutated
#25	IGHV1-69	productive	96.18% (277/288 nt)	IGHJ4	IGHD3-22	[8.8.17]	CARGTSAAGDNAGYYFFYW	Mutated
	IGHV1-69	productive	95.43% (209/219 nt)	IGHJ4	IGHD3-22	[8.8.17]	CARGTSAAGDNAGYYFFYW	Mutated
#26	IGHV4-59	productive	100.00% (285/285 nt)	IGHJ6	IGHD3-22	[8.7.26]	CARAIGGDYYDSSGYYYVNYYYYGMDVW	Unmutated
	IGHV4-59	productive	100.00% (220/220 nt)	IGHJ6	IGHD3-22	[8.7.26]	CARAIGGDYYDSSGYYYVNYYYYGMDVW	Unmutated
#27	IGHV1-2	productive	100.00% (288/288 nt)	IGHJ4	IGHD6-19	[8.8.14]	CARAQWLALGDYFDYW	Unmutated
	IGHV1-2	productive	100.00% (223/223 nt)	IGHJ4	IGHD6-19	[8.8.14]	CARAQWLALGDYFDYW	Unmutated
#28	IGHV1-69	productive	100.00% (288/288 nt)	IGHJ5	IGHD3-3	[8.8.20]	CAREAEDLRFLEWSSSNWFDPW	Unmutated
	IGHV1-69	productive	100.00% (219/219 nt)	IGHJ5	IGHD3-3	[8.8.20]	CAREAEDLRFLEWSSSNWFDPW	Unmutated
#29	IGHV3-11	productive	97.92% (282/288 nt)	IGHJ4	IGHD5-24	[8.8.13]	CATPQTGDGSPFDYW	Mutated (Border line)
	IGHV3-11	productive	97.76% (218/223 nt)	IGHJ4	IGHD5-24	[8.8.13]	CATPQTGDGSPFDYW	Mutated (Border line)
#30	IGHV4-39	productive	100.00% (291/291 nt)	IGHJ4	IGHD1-1	[10.7.13]	CARLPRWNDDTFDYW	Unmutated
	IGHV4-39	productive	100.00% (226/226 nt)	IGHJ4	IGHD1-1	[10.7.13]	CARLPRWNDDTFDYW	Unmutated
#31	IGHV5-51	productive	94.83% (276/288 nt)	IGHJ5	IGHD4-17	[8.8.21]	CARRELDSGDYMFTLEHHCFDPW	Mutated
	IGHV5-51	productive	94.59% (210/222 nt)	IGHJ5	IGHD4-17	[8.8.21]	CARRELDSGDYMFTLEHHCFDPW	Mutated
#32	IGHV6-1	productive	96.96% (288/297 nt)	IGHJ5	IGHD6-19	[10.9.14]	CARVTSISGWYVPDPW	Mutated
	IGHV6-1	productive	96.52% (222/230 nt)	IGHJ5	IGHD6-19	[10.9.14]	CARVTSISGWYVPDPW	Mutated
#33	IGHV4-4	productive	89.12% (254/285 nt)	IGHJ4	IGHD3-3	[8.7.11]	CARDPFQSSFDFW	Mutated
	IGHV4-4	productive	90.00% (198/220 nt)	IGHJ4	IGHD3-3	[8.7.11]	CARDPFQSSFDFW	Mutated
#34	IGHV3-53	productive	99.03% (279/285 nt)	IGHJ6	IGHD2-2	[8.7.8]	CARDVPVDVW	Unmutated
	IGHV3-53	productive	98.64% (217/220 nt)	IGHJ6	IGHD2-2	[8.7.8]	CARDVPVDVW	Unmutated
#35	IGHV4-34	productive	95.79% (273/285 nt)	IGHJ4	IGHD3-10	[7.7.16]	CARTFDYDDLSYFYLAYW	Mutated
	IGHV4-34	productive	94.55% (208/220 nt)	IGHJ4	IGHD3-10	[8.7.16]	CARTFDYDDLSYFYLAYW	Mutated
#36	IGHV3-30	unproductive	100.00% (288/288 nt)	IGHJ4	IGHD3-22	[8.8.15]	CAKDRHPLL***WWVYW	Unmutated
	IGHV1-69	productive	100.00% (28/288 nt)	IGHJ3	IGHD3-16	[8.8.21]	CARGGNYDYVWGSYRPNDAFDIW	
	IGHV3-30	unproductive	100.00% (222/222 nt)	IGHJ4	IGHD3-22	[8.8.15]	CAKDRHPLL***WWVYW	Unmutated
	IGHV1-69	productive	100.00% (219/219 nt)	IGHJ3	IGHD3-16	[8.8.21]	CARGGNYDYVWGSYRPNDAFDIW	

Highligthed grey: Leaders Primer; Not highlighted: FR1 primers

Supplementary table 3

Patient	*V-GENE and allele*	*V-DOMAIN Functionality*	*V-REGION identity % (nt)*	*J-GENE and allele*	*D-GENE and allele*	*CDR-IMGT lengths*	*AA JUNCTION*
#01	IGHV3-21	productive	99.09% (217/219 nt)	IGHJ4	IGHD3-22	[8.8.12]	CARPSDDSSMFDYW
	IGHV3-21	productive	99.10% (221/223 nt)	IGHJ4	IGHD3-22	[8.8.12]	CARPSDDSSMFDYW
#02	IGHV1-46	productive	88.00% (198/225 nt)	IGHJ4	IGHD1-26	[8.8.12]	CARRSNTKGDFDYW
	IGHV1-46	productive	87.95% (197/224 nt)	IGHJ4	IGHD1-26	[8.8.12]	CARRSNTKGDFDYW
#03	IGHV5-51	productive	99.55% (221/222 nt)	IGHJ6	IGHD6-19	[8.8.19]	CARRSDPNGGWSYYYYGMDVW
	IGHV5-51	productive	99.65% (221/223 nt)	IGHJ6	IGHD6-19	[8.8.19]	CARRSDPNGGWSYYYYGMDVW
	IGHV4-34	productive	98.18% (216/220 nt)	IGHJ4	IGHD4-23	[8.7.13]	CAKGGRASLYYFDYW
#04	NA	NA	NA	NA	NA	NA	NA
	IGHV4-39	productive	100.00% (226/226 nt)	IGHJ6	IGHD2-2	[10.7.22]	CARDRGGYCSSTSCYYYYYGMDVW
#05	IGHV1-2	unproductive	89.69% (200/223 nt)	IGHJ4	IGHD1-26	[8.8.X]	SFSNSENFLL#
	IGHV1-2	unproductive	89.38% (202/226 nt)	IGHJ1	IGHD1-26	[8.8.9]	SFSNSENFLLR
	IGHV3-23	productive	94.17% (210/223 nt) [93.72% (209/223 nt)]	IGHJ4	IGHD5-24	[8.8.10]	CAKGRDGYIHRW
	IGHV3-23	productive	82.30% (186/226 nt) [80.08% (181/226 nt)]	IGHJ4…	IGHD5-24	[8.8.10]	CAKGRDGYIHRW
#06	IGHV3-7	unproductive	100.00% (217/217 nt)	IGHJ4	IGHD6-19	[8.8.X]	CARLLVGPSDKGIAVAD#FDYW
	IGHV3-7	unproductive	100.00% (215/215 nt)	IGHJ4	IGHD6-19	[8.8.X]	CARLLVGPSDKGIAVAD#FDYW
	IGHV5-51	productive	100.00% (225/225 nt)	IGHJ4	IGHD3-22	[8.8.14]	CARRQTWTLVPHYDYW
	IGHV5-51	productive	100.00%(228/228 nt)	IGHJ4	IGHD3-22	[8.8.14]	CARRQTWTLVPHYDYW
#07	IGHV2-5	productive	99.20% (247/249 nt)	IGHJ5	IGHD3-3	[10.7.23]	CAHTLQTYYDFWSGQPRRWGWFDPW
	IGHV2-5	productive	99.60% (248/249 nt)	IGHJ5	IGHD3-3	[10.7.23]	CAHTLQTYYDFWSGQPRRWGWFDPW
#08	IGHV4-34	productive	91.40% (202/221 nt)	IGHJ4	IGHD2-21	[8.7.16]	CARGNCGGDCYFSPFDSW
	IGHV4-34	productive	91.32% (200/219 nt)	IGHJ4	IGHD2-21	[8.7.16]	CARGNCGGDCYFSPFDSW
#09	IGHV3-48	productive	89.08% (204/229 nt) [88.65% (203/229 nt)]	IGHJ4	IGHD1-26	[8.8.7]	CARGLGDYW
	IGHV3-48	productive	88.79% (198/223 nt) [88.34% (197/223 nt)]	IGHJ4	IGHD1-26	[8.8.7]	CARGLGDYW
#10	IGHV1-3	productive	90.87% (199/219 nt)	IGHJ4	IGHD6-6	[8.8.17]	CARDWGVSSSPKWEHFDYW
	IGHV1-3	productive	90.87% (199/219 nt)	IGHJ4	IGHD6-6	[8.8.17]	CARDWGVSSSPKWEHFDYW
	IGHV2-5	productive	93.98% (234/249 nt)	IGHJ1	IGHD6-6	[10.7.14]	CAHRDYSTSSGYFQHW
	IGHV2-5	productive	93.98% (234/249 nt)	IGHJ1	IGHD6-6	[10.7.14]	CAHRDYSTSSGYFQHW
#11	IGHV1-2	productive	100.00% (219/219 nt)	IGHJ6	IGHD3-9	[8.8.22]	CAREYGVDILTGYLLLCTYGMDVW
	IGHV1-2	productive	100.00% (219/219 nt)	IGHJ6	IGHD3-9	[8.8.22]	CAREYGVDILTGYLLLCTYGMDVW
	IGHV3-11	unproductive	100.00% (215/215 nt)	IGHJ4	IGHD3-22	[8.8.24]	CARARVEP*PEL***W*KNPTPFDYW
	IGHV3-11	unproductive	100.00% (223/223 nt)	IGHJ4	IGHD3-22	[8.8.24]	CARARVEP*PEL***W*KNPTPFDYW
#12	IGHV6-1	productive	95.12% (234/246 nt)	IGHJ4	IGHD1-1	[10.9.12]	CVRDPTNWSYFDYW
	IGHV6-1	productive	94.78% (218/230 nt)	IGHJ4	IGHD1-1	[10.9.12]	CVRDPTNWSYFDYW
#13	IGHV3-49	productive	95.18% (217/228 nt) [94.74% (216/228 nt)]	IGHJ1	IGHD3-22	[8.9.16]	CTRSYDGSGYYPGYFLLW
	IGHV3-49	productive	95.13% (215/226 nt) [94.69% (214/226 nt)]	IGHJ1	IGHD3-22	[8.9.16]	CTRSYDGSGYYPGYFLLW
	IGHV4-34	productive	92.73% (204/220 nt)	IGHJ6	IGHD3-22	[8.7.20]	CARVSFEGSYDISGYNYYMDVW
#14	IGHV3-7	productive	93.45% (214/229 nt)	IGHJ4	IGHD6-19	[8.8.14]	CATYRETSGWYALDSW
	IGHV3-7	productive	93.24% (208/223 nt)	IGHJ4	IGHD6-19	[8.8.14]	CATYRETSGWYALDSW
#15	IGHV3-9	productive	100.00% (223/223 nt)	IGHJ6	IGHD2-15	[8.8.25]	CAKEFRGGWYYFPPPSHHYYYYGMDVW
	IGHV3-9	productive	100.00% (223/223 nt)	IGHJ6	IGHD2-15	[8.8.25]	CAKEFRGGWYYFPPPSHHYYYYGMDVW
	IGHV4-59 †	productive	95.91% (211/220 nt)	IGHJ2	IGHD2-15	[8.7.18]	CARDRYCSGGTCFDWYFDLW
#16	IGHV3-30	productive	100.00% (229/229 nt)	IGHJ4	IGHD6-19	[8.8.12]	CARDPSSGWYLDYW
	IGHV3-30	productive	100.00% (223/223 nt)	IGHJ4	IGHD6-19	[8.8.12]	CARDPSSGWYLDYW
	IGHV4-34	unproductive	100.00% (220/220 nt)	IGHJ4	IGHD3-16	[8.7.X]	CASINDYVWGSYR*P#YYFDYW
	IGHV4-34	unproductive	100.00% (220/220 nt)	IGHJ4	IGHD3-16	[8.7.X]	CASINDYVWGSYR*P#YYFDYW
#17	IGHV3-53	productive	95.91% (211/220 nt) [95.45% (210/220 nt)]	IGHJ6	IGHD3-9	[8.7.21]	CAREQQDYNLSTGSYFYYGMDVC
	IGHV3-53	productive	95.91% (211/220 nt) [95.45% (210/220 nt)]	IGHJ6	IGHD3-9	[8.7.21]	CAREQQDYNLSTGSYFYYGMDVC
#18	IGHV1-2	unproductive	100.00% (219/219 nt)	IGHJ4	IGHD2-2	[8.8.X]	CARASCSSTSY*R#FDYW
	IGHV1-2	unproductive	100.00% (219/219 nt)	IGHJ4	IGHD2-2	[8.8.X]	CARASCSSTSY*R#FDYW
	IGHV3-30	productive	100.00% (223/223 nt)	IGHJ6	IGHD5-18	[8.8.24]	CARDQDSFFHLADVDTAMSDYGMDVW
#19	IGHV4-31	productive	93.13% (217/233 nt)	IGHJ4	IGHD2-8	[10.7.11]	CAREMYGSSFDYW
	IGHV4-31	productive	92.89% (209/225 nt)	IGHJ4	IGHD2-8	[10.7.11]	CAREMYGSSFDYW
	IGHV3-23 †	productive	99.12% (225/227 nt)	IGHJ4	IGHD3-10	[8.8.10]	CAKDRGDYFDYW

#20	IGHV1-3	productive	100.00% (219/219 nt)	IGHJ6	IGHD1-26	[8.8.17]	CARMYRGSYYYYYYGMDVW
	IGHV1-3	productive	100.00% (219/219 nt)	IGHJ6	IGHD1-26	[8.8.17]	CARMYRGSYYYYYYGMDVW
#21	IGHV6-1	productive	93.97% (218/232 nt)	IGHJ5	IGHD1-26	[10.9.19]	CARGGVGATERTRRKNWFDPW
	IGHV6-1	productive	93.78% (211/225 nt)	IGHJ5	IGHD1-26	[10.9.19]	CARGGVGATERTRRKNWFDPW
#22	IGHV1-NL1	unproductive	89.91% (196/218 nt) [89.45% (195/218 nt)]	IGHJ6	IGHD3-3	[8.8.X]	CVRDP*NAIFGVVI*#YYCGMDVW
	IGHV1-NL1	unproductive	89.91% (196/218 nt) [89.45% (195/218 nt)]	IGHJ6	IGHD3-3	[8.8.X]	CVRDP*NAIFGVVI*#YYCGMDVW
	IGHV3-15	productive	94.04% (221/235 nt)	IGHJ3	IGHD2-15	[8.10.11]	CARGGGGGAWGIW
	IGHV3-15	productive	93.89% (215/229 nt)	IGHJ3	IGHD2-15	[8.10.11]	CARGGGGGAWGIW
#23	IGHV4-59	productive	100.00% (226/226 nt)	IGHJ6	IGHD5-24	[8.7.17]	CARDGPIDTFYYYYGMDVW
	IGHV4-59	productive	100.00% (220/220 nt)	IGHJ6	IGHD5-24	[8.7.17]	CARDGPIDTFYYYYGMDVW
#24	IGHV5-10-1	productive	100.00% (228/228 nt)	IGHJ4	IGHD6-19	[8.8.13]	CARVQWLGLEYFDYW
	IGHV5-10-1	productive	100.00% (222/222 nt)	IGHJ4	IGHD6-19	[8.8.13]	CARVQWLGLEYFDYW
	IGHV3-30 †	productive	96.44% (217/225 nt)	IGHJ4	IGHD3-22	[8.8.17]	CANHLYDSSGYYQNAIDYW
#25	IGHV1-69	productive	95.52% (213/223 nt)	IGHJ4	IGHD3-22	[8.8.17]	CARGTSAAGDNAGYYFFYW
	IGHV1-69	productive	95.43% (209/219 nt)	IGHJ4	IGHD3-22	[8.8.17]	CARGTSAAGDNAGYYFFYW
#26	IGHV4-59	productive	100.00% (220/220 nt)	IGHJ6	IGHD3-22	[8.7.26]	CARAIGGDYYDSSGYYYVNYYYYGMDVW
	IGHV4-59	productive	100.00% (220/220 nt)	IGHJ6	IGHD3-22	[8.7.26]	CARAIGGDYYDSSGYYYVNYYYYGMDVW
#27	IGHV1-2	productive	100.00% (219/219 nt)	IGHJ4	IGHD6-19	[8.8.14]	CARAQWLALGDYFDYW
	IGHV1-2	productive	100.00% (223/223 nt)	IGHJ4	IGHD6-19	[8.8.14]	CARAQWLALGDYFDYW
#28	IGHV1-69	productive	100.00% (225/225 nt)	IGHJ5	IGHD3-3	[8.8.20]	CAREAEDLRFLEWSSSNWFDPW
	IGHV1-69	productive	100.00% (219/219 nt)	IGHJ5	IGHD3-3	[8.8.20]	CAREAEDLRFLEWSSSNWFDPW
#29	IGHV3-11	productive	97.80% (222/227 nt)	IGHJ4	IGHD5-24	[8.8.13]	CATPQTGDGSPFDYW
	IGHV3-11	productive	97.76% (218/223 nt)	IGHJ4	IGHD5-24	[8.8.13]	CATPQTGDGSPFDYW
#30	IGHV4-39	productive	100.00% (234/234 nt)	IGHJ4	IGHD1-1	[10.7.13]	CARLPRWNDDTFDYW
	IGHV4-39	productive	100.00% (226/226 nt)	IGHJ4	IGHD1-1	[10.7.13]	CARLPRWNDDTFDYW
#31	IGHV5-51	productive	94.59% (210/222 nt)	IGHJ5	IGHD4-17	[8.8.21]	CARRELDSGDYMFTLEHHCFDPW
	IGHV5-51	productive	94.59% (210/222 nt)	IGHJ5	IGHD4-17	[8.8.21]	CARRELDSGDYMFTLEHHCFDPW
#32	IGHV6-1	productive	96.21% (203/211 nt)	IGHJ5	IGHD6-19	[6.9.14]	CARVTSISGWYVPDPW
	IGHV6-1	productive	96.52% (222/230 nt)	IGHJ5	IGHD6-19	[10.9.14]	CARVTSISGWYVPDPW
#33	IGHV4-4	productive	90.35% (206/228 nt)	IGHJ4	IGHD3-3	[8.7.11]	CARDPFQSSFDFW
	IGHV4-4	productive	90.00% (198/220 nt)	IGHJ4	IGHD3-3	[8.7.11]	CARDPFQSSFDFW
	IGHV3-9 ‡	productive	100.00% (223/223 nt)	IGHJ6	IGHD2-15	[8.8.25]	CAKEFRGGWYYFPPPSHHYYYYGMDVW
#34	IGHV3-53	productive	98.11% (208/212 nt)	IGHJ6	IGHD2-2	[8.7.8]	CARDVPVDVW
	IGHV3-53	productive	98.64% (217/220 nt)	IGHJ6	IGHD2-2	[8.7.8]	CARDVPVDVW
	IGHV1-69 †	unproductive	97.76% (218/223 nt)	IGHJ4	IGHD3-22	[8.8.18]	CAGPG*YYDSGGPNKRGDYW
#35	IGHV4-34	productive	94.20% (195/207 nt)	IGHJ4	IGHD3-10	[7.7.16]	CARTFDYDDLSYFYLAYW
	IGHV4-34	productive	94.55% (208/220 nt)	IGHJ4	IGHD3-10	[8.7.16]	CARTFDYDDLSYFYLAYW
#36	IGHV3-30	unproductive	100.00% (242/242 nt)	IGHJ4	IGHD3-22	[8.8.15]	CAKDRHPLL***WWVYW
	IGHV3-30	unproductive	100.00% (222/222 nt)	IGHJ4	IGHD3-22	[8.8.15]	CAKDRHPLL***WWVYW
	IGHV1-69	productive	100.00% (219/219 nt)	IGHJ3	IGHD3-16	[8.8.21]	CARGGNYDYVWGSYRPNDAFDIW

Highligthed grey: MinION sequencing; Not highlighted: Sanger Method; † Validated by alternative PCR; ‡ Not validated by alternative PCR
